# Preclinical Cerebral Network Connectivity Evidence of Deficits in Mild White Matter Lesions

**DOI:** 10.3389/fnagi.2016.00027

**Published:** 2016-02-18

**Authors:** Ying Liang, Xuan Sun, Shijun Xu, Yaou Liu, Ruiwang Huang, Jianjun Jia, Zhanjun Zhang

**Affiliations:** ^1^State Key Laboratory of Cognitive Neuroscience and Learning and IDG/McGovern Institute for Brain Research, Beijing Normal UniversityBeijing, China; ^2^BABRI Centre, Beijing Normal UniversityBeijing, China; ^3^Department of Geriatric Neurology, Chinese PLA General HospitalBeijing, China; ^4^Chengdu University of Traditional Chinese MedicineChengdu, China; ^5^Department of Radiology, Xuanwu Hospital, Capital Medical UniversityBeijing, China; ^6^Centre for Studies of Psychological Application, South China Normal UniversityGuangzhou, China

**Keywords:** mild white matter lesions, resting-state networks, functional connectivity, white matter integrity, cognitive impairments

## Abstract

White matter lesions (WMLs) are notable for their high prevalence and have been demonstrated to be a potential neuroimaging biomarker of early diagnosis of Alzheimer’s disease. This study aimed to identify the brain functional and structural mechanisms underlying cognitive decline observed in mild WMLs. Multi-domain cognitive tests, as well as resting-state, diffusion tensor and structural images were obtained on 42 mild WMLs and 42 age/sex-matched healthy controls. For each participant, we examined the functional connectivity (FC) of three resting-state networks (RSNs) related to the changed cognitive domains: the default mode network (DMN) and the bilateral fronto-parietal network (FPN). We also performed voxel-based morphometry analysis to compare whole-brain gray matter (GM) volume, atlas-based quantification of the white matter tracts interconnecting the RSNs, and the relationship between FC and structural connectivity. We observed FC alterations in the DMN and the right FPN combined with related white matter integrity disruption in mild WMLs. However, no significant GM atrophy difference was found. Furthermore, the right precuneus FC in the DMN exhibited a significantly negative correlation with the memory test scores. Our study suggests that in mild WMLs, dysfunction of RSNs might be a consequence of decreased white matter structural connectivity, which further affects cognitive performance.

## Introduction

White matter lesions (WMLs) are present in over 50% of elderly people as previously reported (Prins and Scheltens, 2015). WMLs are widely recognized as a cerebral small-vessel disease, which result in ischemic brain impairments (Schmidt et al., 2011), and are associated with stroke, dementia, disability, and even death (Poggesi et al., 2010). Some studies even reported that WMLs may predict the risk of Alzheimer’s disease (AD; Silbert et al., 2012; Brickman et al., 2015). WMLs can be graded as mild, moderate, and severe using several visual rating scales, one of the most common is the age-related WM changes (ARWMC) rating scale (Wahlund et al., 2001; Bocti et al., 2005). However, the underlying neural mechanism of how WMLs affect the cognition is still controversial. One possible reason is, most studies have focused on severe WMLs currently, while the mild WMLs (mWMLs) have long been neglected due to its insidious onset and unnoticeable cognitive decline in early stages (de Leeuw et al., 2002). There are a lot of confounding factors in severe WMLs, like stroke, vascular dementia and AD. One can hardly clarify if the effects are from these diseases or WMLs. However, we argue the effect of mWMLs on health should not be underestimated. With the relatively high prevalence of mWMLs in the aged population, ranging from 8.7 to 33% (Launer et al., 2005; Zhou et al., 2008), it may also offer a relatively “pure” model to study WMLs, and should be given attention in clinical practice.

Recent studies suggested that specific cognitive functions rely on different resting-state networks (RSNs), such as default mode network (DMN) and fronto-parietal network (FPN; Olesen et al., 2003; Greicius et al., 2004; Bressler and Menon, 2010). DMN is associated with episodic memory while FPN support executive functions, and disconnection among these RSNs accounted for related cognitive deficits (Olesen et al., 2003; Greicius et al., 2004). As anatomical foundations, the underlying structural functional connectivity (FC) in the brain is widely recognized for its important roles. Indeed, emerging data revealed that structural connections are highly predictive of functional connections (Honey et al., 2009). For example, Greicius et al. (2009) demonstrated that white matter (WM) fibers linked components of the DMN. Olesen et al. (2003) also showed that maturation of the structural substrate supports normal function of the FPN.

However, to the best of our knowledge, there are few studies that have investigated the intrinsic functional changes due to mWMLs, as well as the interaction between structure and function in mWMLs. In this study, we hypothesized that (1) mWMLs have cognitive impairment in certain domains; (2) a diffuse alteration of RSN FC may serve as a substrate of the declined cognitive domains, and (3) microstructural WM changes affect the RSNs. To test our hypotheses, we examined the cognitive functions, brain functional networks, and brain structures mWMLs, as well as the relationship between them.

## Materials and Methods

### Participants

Subjects were recruited from the Beijing Aging Brain Rejuvenation Initiative (BABRI) Study, which is a longitudinal study investigating aging and cognitive impairment in urban elderly individuals in Beijing, China. The present study used the first time data. All magnetic resonance imaging (MRI) data quality at this time were checked by the BABRI staff before finally including into the BABRI database. Two hundred and fourteen subjects who had T2-weighted Fluid-attenuated inversion recovery (T2w-FLAIR) images were diagnosed by an experienced rater who was blind to the clinical data for twice, each on different days. Only subjects who were diagnosed as mild WMLs at both times were included into the mild WMLs database to ensure an intrarater reliability of 100%. Fifty-one subjects were diagnosed as mWMLs at both times. We excluded four subjects aged over 80 years, and five whose multimodal MRI data were not complete. Then 42 subjects with mWMLs and 42 socio-demographically matched healthy elderly controls from BABRI healthy elderly database (aged 50–80 years, education > 6 years) were included in this study. All subjects were right-handed and native Chinese speakers. In order to avoid confounding factors from brain lesions, we set the exclusive criteria as: (1) no structural abnormalities, such as tumors, subdural hematomas, or contusions due to previous head trauma; (2) no history of addiction, neurological or psychiatric diseases; (3) no conditions known to affect cerebral function, including alcoholism, current depression, Parkinson’s disease, or epilepsy; and (4) no large vessel disease such as cortical or subcortical infarcts and watershed infarcts. Demographic information for each group is presented in **Table 1**. This study was approved by the Institutional Review Board of the Beijing Normal University Imaging Center for Brain Research. Written informed consent was obtained from each subject prior to the MRI scanning.

**Table 1:** Demographics of all subjects, and neuropsychological test results.

	Controls (*n* = 42)	mWMLs (*n* = 42)	*T*-value (*X*^2^)	*p*-value
Age (years)	64.5 ± 6.6	63.2 ± 6.5	0.93	0.35
Education (years)	10.0 ± 3.2	10.3 ± 3.2	0.44	0.66
Sex (M/F)	15/27	13/29	0.21	0.82
General mental status				
MMSE	26.4 ± 2.5	27.1 ± 1.8	1.39	NS
Episodic memory				
AVLT	3.4 ± 2.6	4.9 ± 2.5	2.73	0.008
ROCF Recall	8.7 ± 6.7	16.5 ± 10.3	4.10	<0.001
Working memory				
Digit Span	11.5 ± 2.1	11.4 ± 2.1	0.16	NS
Processing speed				
Digit Symb-Coding	28.6 ± 9.4	32.5 ± 11.6	1.72	NS
Executive function				
TMT-BA time	144.3 ± 73.2	131.5 ± 78.7	0.77	NS
Stroop CB	44.1 ± 20.3	34.6 ± 15.3	2.43	0.017
Reasoning				
Similarities	14.7 ± 3.7	15.1 ± 4.4	0.48	NS
Language ability				
BNT	23.0 ± 3.3	22.0 ± 5.2	1.08	NS

*The p-value for sex distribution in the two groups was obtained using a Chi-square test.*

### Cognitive Assessment

All subjects underwent a battery of neuropsychological tests to assess cognitive functions. General mental status was assessed using the Mini-Mental State Examination (MMSE). Processing speed was assessed using the Digit Symbol-Coding subtest of the Wechsler Adult Intelligence Scale-Revised Chinese revision (WAIS-RC; Gong, 1992). Verbal and non-verbal episodic memory tests included the Auditory Verbal Learning Test (AVLT; Guo, 2007) and the Recall component of the Rey–Osterrieth Complex Figure Test (ROCF; Zhou et al., 2006). Executive function was assessed using the Stroop Test (Guo et al., 2005) and Trail Making Test (TMT; Lu et al., 2006). Verbal working memory was assessed using the Digit Span scores on the WAIS-RC. Verbal reasoning and abstract thinking were assessed using the Similarities subtest of the WAIS-RC. Lastly, language ability was assessed using the Boston Naming Test (BNT; Guo et al., 2006). Neuropsychological characterizations for each group are presented in **Table 1**.

### Image Acquisition

All subjects were scanned using a Siemens Trio 3T MRI scanner in the Imaging Center for Brain Research at Beijing Normal University. Diffusion tensor images were acquired using a twice-refocused spin-echo diffusion-weighted EPI sequence with the following parameters (70 axial sections, section thickness [ST] = 2 mm, no section gap, 30 diffusion directions with a *b*-value of 1000 s/mm^2^ and an additional image with a *b*-value of 0 s/mm^2^, field of view [FOV] = 256 mm × 256 mm, acquisition matrix [AM] = 128 × 128, number of signals acquired = 3). Resting-state fMRI (rs-fMRI) data were acquired using a single-shot gradient echo-planar imaging (EPI) sequence (33 axial slices, repetition time [TR] = 2000 ms, echo time [TE] = 30 ms, ST = 3.5 mm, flip angle [FA] = 90°, FOV = 200 mm × 200 mm, AM = 64 × 64, slice gap = 0.7 mm). Subjects were instructed to stay awake, relax with their eyes closed and remain as motionless as possible during resting fMRI scan. The resting scan lasted for 8 min and 240 image volumes were obtained. T1-weighted brain structural images were acquired using 3D magnetization prepared rapid gradient echo (MP-RAGE) sequence (176 sagittal slices, TR = 1900 ms, TE = 3.44 ms, ST = 1 mm, FA = 9°, FOV = 256 mm × 256 mm, AM = 256 × 256). A T2w-FLAIR sequence was also applied to measure WM hyperintensities.

### Diagnosis of mWMLs

All subjects were examined using the T2w-FLAIR pulse sequence. The deficit severity of WMLs were rated based on axial T2w-FLAIR images using the ARWMC rating scale (Wahlund et al., 2001). Briefly, WMLs were rated separately on a four-point scale (0, no lesions; 1, punctuate; 2, beginning confluence; 3, diffuse involvement of the entire region) in five regions (frontal, parieto-occipital, temporal, and infra-tentorial) in the left and right hemispheres, resulting in an ARWMC sum score of 0 to 30. Subjects were also graded as having a mild (1 to 4 points), moderate (5 to 8 points), or severe (≥9 point) WMLs. All ratings were performed by an experienced rater who was blind to the clinical data for twice, each on different days. The controls showed no evidence of WMLs on FLAIR MRIs.

### Rs-fMRI Preprocessing and Analysis

Image preprocessing and analysis were performed using Statistical Parametric Mapping (SPM8^1^) and the Data Processing Assistant for Resting-State fMRI (DPARSF; Chao-Gan and Yu-Feng, 2010), including slice timing, realignment, spatial normalization, resampling, spatial smoothing, and high-pass temporal filtering. Then we performed the independent component analysis (ICA) using the group ICA toolbox (GIFT version 2.0e^2^). The best-fit components for the DMN and bilateral FPN were identified by visual inspection. Group differences in regional FC were identified through SPM8 using an analysis of covariance (ANCOVA) model controlling for age, gender and education [*p* < 0.05, false discovery rate (FDR) corrected; cluster size > 20 voxels].

### Structural Image Analysis

We used a voxel-based morphometry (VBM) analysis using FMRIB Software Library (FSL^3^) Tools to compare whole-brain gray matter (GM) volume difference between the mWMLs and the controls.

### Diffusion Tensor Imaging Preprocessing and Atlas-Based Quantification of the Selected Tracts

Diffusion tensor imaging (DTI) data preprocessing were performed using FMRIB’s Diffusion Toolbox (FDT; FSL 4.1.4^4^). We first corrected the original data for the effects of head movement and eddy currents. Then, we created a brain mask by running the BET procedure on the b0 (no diffusion weighting) images. At last, we determined voxel-wise maps of fractional anisotropy (FA) and mean diffusivity (MD) by fitting the diffusion tensor model.

Many studies have suggested that the brain regions of RSNs are anatomically connected through WM tracts (Honey et al., 2009). The DMN contains the following tracts: the cingulum bundle (CB), inferior cingulum bundle (ICB), bilateral superior frontal occipital fasciculus (SFOF), and corpus callosum (CC; Greicius et al., 2009; Teipel et al., 2010; Sharp et al., 2011). The superior longitudinal fasciculus (SLF) was suggested to interconnect the FPN (Turken et al., 2008; van den Heuvel et al., 2009). To investigate the diffusion changes in these specific tracts, we adopted the atlas-based segmentation strategy. Average values of FA and MD were computed for major tracts in each subject. We selected the widely used digital WM atlas JHU ICBM-DTI-81^5^ .

### Statistical Analysis

#### Cognitive Assessment

The significance of group differences in age, education, and neuropsychological scores were examined using two-sample *t*-tests. Gender data were analyzed using a χ^2^-test.

#### Diffusion Tensor Imaging

Analysis of covariance was used to determine the significant altered FA and MD between groups for each of the atlas-based tract ROIs. In the calculation, we regressed out the effects of age, gender, and education. Bonferroni correction was used to adjust for potential spurious findings due to the number of ROIs examined.

#### Correlation of RSN and Structural Connectivity

For each network, multiple regression analyses were performed to assess the relationship between the individual FC maps and diffusion metrics as well as the neuropsychological tests in mWMLs. The tests were masked by the map of each RSN in mWMLs. The statistical threshold was set at *p* < 0.005 (not corrected) at the voxel-level (controlled for age, gender, and education). We also did the same analyses in control group.

## Results

### Demographic and Cognitive Characteristics

Statistical comparison showed no significantly differences in age, in sex, and in education between the controls and the mWMLs. Scores on the all episodic memory tests (AVLT: *p* = 0.008, ROCF recall: *p* < 0.001) and Stroop CB (*p* = 0.017) were significantly decreased in the mWMLs compared to the healthy controls (**Table 1**).

### RSN Abnormalities

**FIGURE 1:**
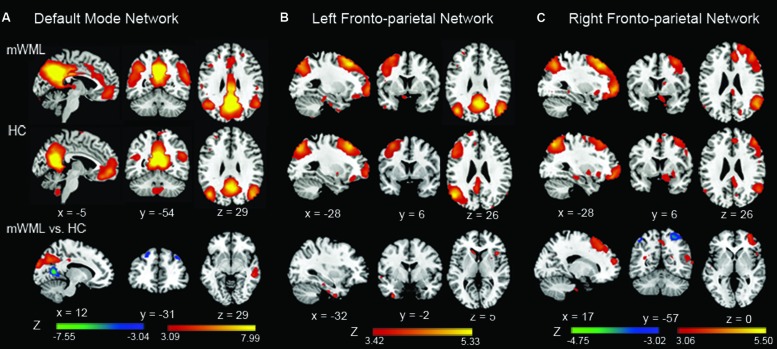
**Spatial maps of the default mode network (DMN) and fronto-parietal network (FPN).** In the DMN **(A)**, mild white matter lesion (WML) patients showed increased connectivity in the precuneus, inferior parietal cortex, and middle temporal compared to healthy controls. The bilateral dorsolateral prefrontal cortex (DLPFC) and left medial prefrontal cortex (MPFC), and calcarine cortex showed a significantly decreased DMN connectivity in patients. In the left FPN **(B)**, no significant difference between the groups was found. In the right FPN **(C)**, mWMLs showed a significantly increased connectivity in the right middle frontal gyrus and dorsolateral prefrontal cortex, and a decreased connectivity in the bilateral superior parietal cortex. The results were displayed at a probability threshold of *p* < 0.05, FDR corrected at the whole brain level. Color bars represent *t*-scores.

Within the DMN, the mWMLs showed a significant increased FC in the bilateral precuneus, bilateral inferior parietal gyrus (IPG) and the right middle temporal gyrus (MTG) relative to the HC. Several frontal areas, including the bilateral dorsolateral prefrontal cortex (DLPFC) and left medial prefrontal cortex (MPFC), and calcarine cortex showed a significantly decreased FC in the mWMLs. Within the left FPN, no FC differences were found between the mWMLs and the controls after FDR correction. When we set the threshold at *p* < 0.001 uncorrected, the mWMLs showed increased FC in the left inferior temporal gyrus (ITG) and right inferior frontal gyrus (IFG). In the right FPN, the mWMLs showed a significantly increased FC in the right middle frontal gyrus (MFG) and DLPFC. In this same network, the mWMLs compared to controls exhibited a decreased FC in the bilateral superior parietal gyrus (SPG; FDR corrected; **Figure 1**; Supplementary Tables SI and SII).

### Group Differences of VBM

No significant difference in regional cerebral gray matter volume between the controls and the mWMLs.

### Group Comparisons of Atlas-Based Tract ROIs

**FIGURE 2:**
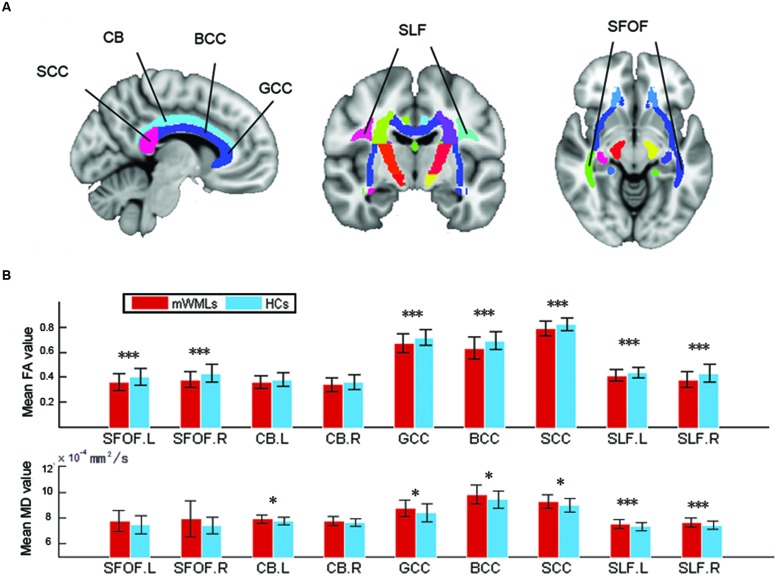
**Mean diffusion metrics of the atlas-based tracts in the mWMLs and control groups. (A)** The JHU-white matter atlas in the ICBM-DTI-81 space. Colored regions indicate related WM tracts involved in the RSNs. FA, fractional anisotropy; MD, mean diffusivity; GCC, genu of corpus callosum; BCC, body of corpus callosum; SCC, splenium of corpus callosum; CB, cingulum bundle at cingulate gyrus; SFOF, superior frontal occipital fasciculus; SLF: superior longitudinal fasciculus. **(B)** Group differences in the mean diffusion metrics of the atlas-based tracts among the mWMLs and HC groups. ^∗^*p* < 0.05; ^∗∗∗^*p* < 0.05 after Bonferroni correction.

Compared to the controls, the mWMLs showed a reduction in FA in the bilateral SFOF, CC, and bilateral SLF (*p* < 0.05 after Bonferroni correction). The SLF also showed an significantly increased MD compared to the controls (**Figure 2**, Supplementary Table SIII).

### Resting-State Networks, WM, and Neuropsychological Score Correlations

**FIGURE 3:**
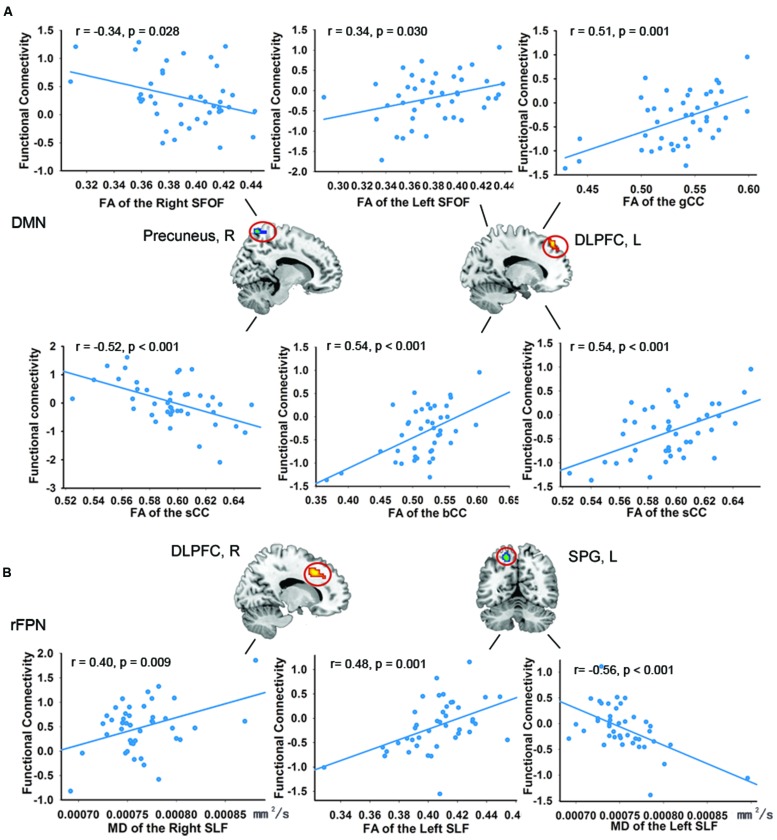
**Correlations between the functional connectivity (FC) of the resting-state networks and structural connectivity. (A)** The significant positive correlations in the DMN. **(B)** The significant positive correlations in the right FPN. The criterion was uncorrected *p* < 0.005 at the voxel level. Colored areas indicate significant regions. The color dots were extracted from the peak correlation voxel in each region.

**FIGURE 4:**
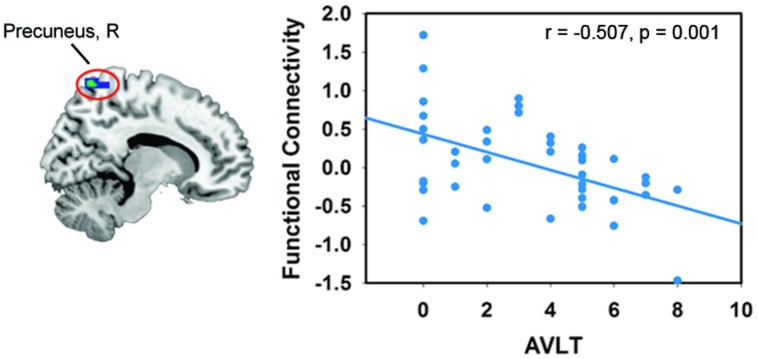
**Correlations between the FC of the resting-state network and neuropsychological score.** The criterion was uncorrected *p* < 0.005 at voxel level. Colored areas indicate significant regions. The color dots were extracted from the peak correlation voxel in each region.

In the DMN, we found significant positive correlations between the *FA*-values in the left SFOF, CC (the genu, body and splenium), and FC in the left DLPFC (*p* < 0.005, uncorrected). The *FA*-values in the right SFOF and splenium of CC were negatively correlated with the FC of right precuneus (**Figure 3**). In addition, the FC of right precuneus in DMN showed a significantly negative correlation with the AVLT score (*r* = –0.507, *p* = 0.001; **Figure 4**). In the right FPN, we found significant correlations between the left SLF and left SPG, as well as the right SLF and right DLPFC. However, we did not find any correlation in the left FPN. The control group only showed marginally significant correlation results in each network (**Supplementary Figure S1**).

## Discussion

Increasing evidence has suggested that a majority of AD patients has concurrent WM changes and WMLs may serve as a neuroimaging biomarker in the context of MCI or AD (Brickman et al., 2015; Prins and Scheltens, 2015). To explore the potential neural mechanism of how WMLs affect the cognition we investigated the DMN and bilateral FPN, as well as their relevant WM tracts. We found FC abnormalities in the DMN, right FPN, and concurrent WM integrity disruption, which engaged in an internal network connection of the DMN and right FPN, respectively. In addition, we also found altered FCs of RSNs correlated with WM fibers and declined cognitive functions. Among these positive findings, there was no significant GM atrophy in mWMLs compared to healthy controls, indicating that an impaired DMN and right FPN might be mainly affected by WM integrity disruption.

Default mode network abnormalities were revealed in a number of AD studies, and some studies have indicated that early detection of DMN changes help to distinguish AD from healthy elderly people (Greicius et al., 2004). The functional disconnections of DMN in our study may reflect the long-term injurious effect of mWMLs and may account for cognitive declines because both the DLPFC and MPFC are involved in cognitive functions, such as the retrieval of episodic memory, executive function, and working memory (Gilmartin et al., 2013). Moreover, enhanced FC in the bilateral IPG, precuneus, and MTG were found in mWMLs. The IPG (Vilberg and Rugg, 2008), MTG (Squire and Zola-Morgan, 1991), and precuneus are involved in maintaining memory function and are the functional hubs in the brain (Buckner et al., 2009). Furthermore, a significant correlation between the precuneus in DMN and episodic memory was also found, which suggested that episodic memory impairment might be a result of enhanced DMN FC. Interestingly, several previous studies have demonstrated similar patterns of FC changes in MCI and AD (Wu et al., 2011).

The FPN is responsible for working memory and attentional executive control (Naghavi and Nyberg, 2005). In our current study, mWMLs showed decreased right FPN FC in the SPG, which has been reported to be related with memory retrieval (Vilberg and Rugg, 2008). Furthermore, increased FC was shown in the frontal areas, which suggested that mWMLs can recruit network resources, primarily from the frontal regions in response to reduced parietal connectivity. One explanation for our right FPN finding is the diverse developmental planning structure of the two hemispheres, resulting in lateralized differences in maturational rates, metabolism, and functional activation (Toga and Thompson, 2003). Moreover, the similarity of our findings to a previous study, which reported that the right lateralized FPN was compromised in AD using resting-state fMRI (Sorg et al., 2007) as well as the right hemi-aging model (Royall et al., 2012).

The brain’s neurophysiological activity was shaped by the structural connectivity together with other factors, and the FC reflects communication between spatially remote brain regions (Wang et al., 2013; Betzel et al., 2014). To understand the potential anatomical foundation of the functional alteration in mWMLs further, we investigated GM volume and WM microstructural differences between the two groups. The abnormal WM bundles in our findings included the bilateral SFOF, CC, and SLF, which were also vulnerable in neurodegenerative diseases like AD (Sexton et al., 2011). However, the VBM results did not show any GM volumetric differences between the groups. So it is conceivable that both microstructural and FC changes may be independent of GM atrophy in the mWMLs.

We further explored the relationship between the changed FC and related structural connectivity. Significant correlations of the RSNs with each of several compromised WM regions were found separately in the DMN and right FPN. These results indicate that structural infrastructure might support functional synchronization between these regions in the RSNs. The abnormal internal brain FC could be affected by extensive aberrant anatomical WM connections. This assumption is supported by the notion that the functional networks are constrained by the topology of the underlying anatomical networks (Honey et al., 2007). The abnormal WM myelin sheath may affect functional communications that rely on highly synchronized neuronal impulses and result in functional “disconnections” of association cortical regions (Bartzokis, 2004). For this reason, we considered that the aberrant FC in the two networks might be at least partly accountable for the structural disconnection.

There are potential limitations to be discussed with our study. The main limitation of our present study was its cross-sectional nature, which limited further investigation on the progression of the relationship between the structural and functional networks in the context of neurodegenerative diseases, such as AD. What’s more, although FC reflects structural connectivity to some extent, the certain structure-function relation might not be transparent. There still need further research to investigate deeper correspondence between structural alternation and changed FC. However, in the correlation analyses, no results were survived after FDR correction. To enlarge the sample size in the future research may increase the statistical power.

## Conclusion

Alterations of both structural and FC were demonstrated in the mWMLs. Our results provided evidence of the structure and functional neural mechanism under cognition in mild WMLs and may offer a better understanding of the WMLs pathomechanism for clinicians. However, there still need further research to investigate deeper correspondence between structural alteration and changed FC.

## Author Contributions

Guarantors of integrity of entire study, ZZ; study concepts/study design, ZZ; data acquisition, all authors; WML diagnosis, YOL, SX; statistical analysis, YL, XS; manuscript drafting or manuscript revision for important intellectual content, all authors; literature research, YL, XS; manuscript editing, RH, JJ, and ZZ; and manuscript final version approval, all authors.

## Conflict of Interest Statement

The authors declare that the research was conducted in the absence of any commercial or financial relationships that could be construed as a potential conflict of interest. The reviewer CC and handling Editor declared their shared affiliation, and the handling Editor states that the process nevertheless met the standards of a fair and objective review.

## Footnotes

1 http://www.fil.ion.ucl.ac.uk/spm

2 http://mialab.mrn.org/software/gift/

3 http://fsl.fmrib.ox.ac.uk/fsl/fslwiki/

4 http://www.fmrib.ox.ac.uk/fsl/FDT

5 http://cmrm.med.jhmi.edu/

## Supplementary material

FIGURE S1 **Correlations between the functional connectivity (FC) of the resting-state networks and structural connectivity in control group**.
