# Supplementary figures and images for: Preclinical Cerebral Network Connectivity Evidence of Deficits in Mild White Matter Lesions
Source: Front Aging Neurosci. 2016 Feb 18;8:27. doi: 10.3389/fnagi.2016.00027 (PMC4757671; doi:10.3389/fnagi.2016.00027)

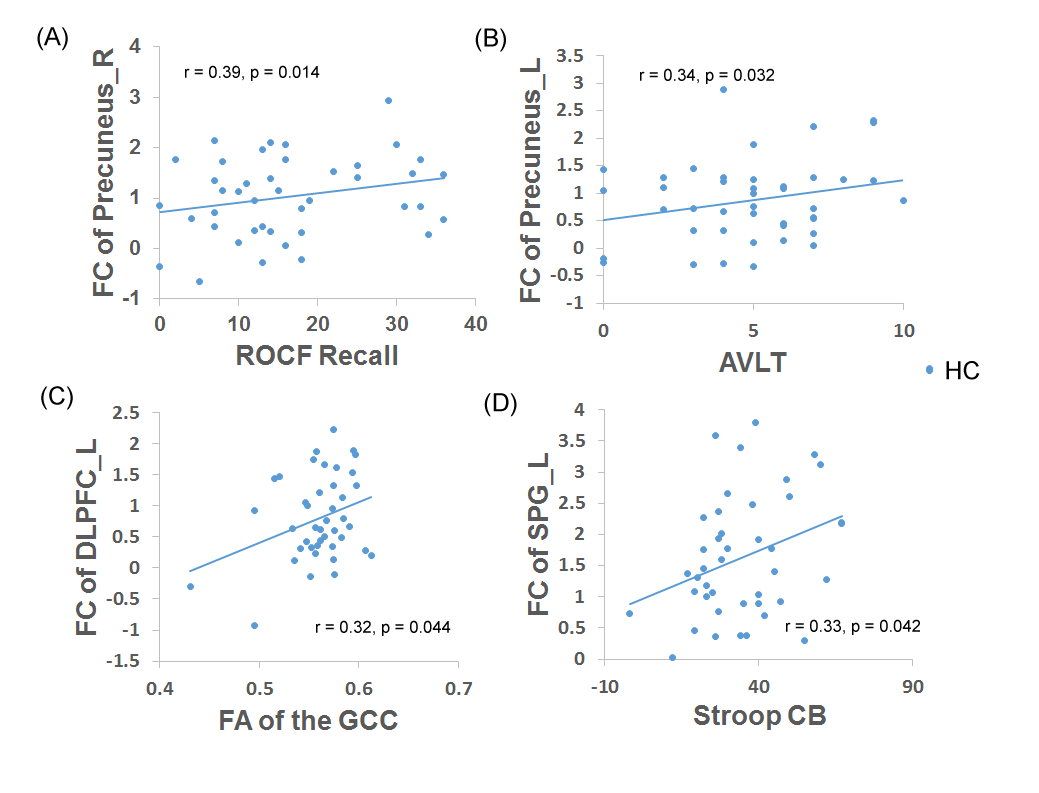

Supplement: FIGURE S1 — Correlations between the functional connectivity (FC) of the resting-state networks and structural connectivity in control group. [file Image_1.TIF]
