# Supplementary material for: Preclinical Cerebral Network Connectivity Evidence of Deficits in Mild White Matter Lesions
Source: Front Aging Neurosci. 2016 Feb 18;8:27. doi: 10.3389/fnagi.2016.00027 (PMC4757671; doi:10.3389/fnagi.2016.00027)
Supplement: Supplementary file 2 [file Table_1.DOC]

**ONLINE SUPPLEMENT**

**Supplemental Table I.** Increased FC clusters in mWMLs compared to controls.

| Region | BA | Cluster size | MNI coordinates | | | Z |
| --- | --- | --- | --- | --- | --- | --- |
|  |  |  | x | y | z |  |
| Default mode network |  |  |  |  |  |  |
| Precuneus, R | 7 | 4202 | 9 | -66 | 36 | 7.93 |
| Precuneus, L | 7 | 195 | -6 | -69 | 39 | 7.61 |
| IPG, L | 39 | 95 | -45 | -60 | 45 | 7.35 |
| IPG, R | 40 | 387 | 51 | -51 | 48 | 5.22 |
| MTG, R | 21 | 340 | 51 | -33 | -6 | 5.50 |
| Fronto-parietal network (left) |  |  |  |  |  |  |
| ITG, L | 20 | 76 | -36 | -3 | -42 | 4.52 |
| IFG, R | 45 | 70 | 42 | 21 | 3 | 4.41 |
| Fronto-parietal network (right) |  |  |  |  |  |  |
| DLPFC, R | 8 | 346 | 21 | 33 | 48 | 5.42 |
| MFG, R | 9 | 346 | 27 | 39 | 42 | 5.32 |

IPG, inferior parietal gyrus; MTG, middle temporal gyrus; ITG, inferior temporal gyrus; IFG, inferior frontal gyrus; DLPFC, dorsolateral prefrontal cortex; MFG, middle frontal gyrus.
