# Supplementary material for: Preclinical Cerebral Network Connectivity Evidence of Deficits in Mild White Matter Lesions
Source: Front Aging Neurosci. 2016 Feb 18;8:27. doi: 10.3389/fnagi.2016.00027 (PMC4757671; doi:10.3389/fnagi.2016.00027)
Supplement: Supplementary file 3 [file Table_2.DOC]

**Supplemental Table II.** Decreased FC clusters in mWMLs compared to controls.

| Region | BA | Cluster size | MNI coordinates | | | Z |
| --- | --- | --- | --- | --- | --- | --- |
|  |  |  | x | y | z |  |
| Default mode network |  |  |  |  |  |  |
| Calcarine | 30 | 551 | -15 | -60 | 9 | 7.55 |
| DLPFC, R | 9 | 199 | 36 | 45 | 36 | 5.39 |
| DLPFC, L | 9 | 116 | -24 | 33 | 48 | 4.69 |
| MPFC, L | 10 | 209 | -39 | 48 | 27 | 4.02 |
| Fronto-parietal network (right) |  |  |  |  |  |  |
| SPG, L | 7 | 124 | -24 | -69 | 57 | 4.75 |
| SPG, R | 7 | 40 | 21 | -60 | 60 | 4.18 |

DLPFC, dorsolateral prefrontal cortex; MPFC, medial prefrontal cortex; SPG, superior parietal gyrus.
