# Supplementary material for: Preclinical Cerebral Network Connectivity Evidence of Deficits in Mild White Matter Lesions
Source: Front Aging Neurosci. 2016 Feb 18;8:27. doi: 10.3389/fnagi.2016.00027 (PMC4757671; doi:10.3389/fnagi.2016.00027)
Supplement: Supplementary file 4 [file Table_3.DOC]

**Supplemental Table III.** Group comparisons of mean FA and MD values of each ROI in mWMLs and controls.

| Tracts | mWMLs | HC | F | *p* |
| --- | --- | --- | --- | --- |
| DMN |  |  |  |  |
| SFOF, FA, L | 0.38±0.03 | 0.40±0.03 | 7.57 | 0.007*** |
| FA,R | 0.39±0.03 | 0.41±0.04 | 10.56 | 0.002*** |
| MD,L | 0.78±0.08 | 0.75±0.07 | 2.67 | NS |
| MD, R | 0.80±0.10 | 0.74±0.07 | 3.71 | 0.058 |
| CB, FA, L | 0.37±0.03 | 0.38±0.03 | 2.68 | NS |
| FA,R | 0.38±0.03 | 0.39±0.03 | 1.29 | NS |
| MD,L | 0.79±0.03 | 0.78±0.03 | 3.95 | 0.050* |
| MD, R | 0.78±0.04 | 0.77±0.02 | 1.14 | NS |
| ICB, FA, L | 0.32±0.03 | 0.33±0.03 | 0.98 | NS |
| FA,R | 0.32±0.03 | 0.33±0.04 | 1.43 | NS |
| MD,L | 0.92±0.12 | 0.90±0.07 | 1.19 | NS |
| MD, R | 0.90±0.12 | 0.88±0.08 | 0.31 | NS |
| GCC, FA | 0.54±0.04 | 0.56±0.03 | 9.24 | 0.003*** |
| MD | 0.88±0.06 | 0.84±0.07 | 5.35 | 0.023* |
| BCC, FA | 0.52±0.04 | 0.55±0.04 | 10.30 | 0.002*** |
| MD | 0.98±0.07 | 0.94±0.06 | 6.56 | 0.012* |
| SCC, FA | 0.60±0.03 | 0.61±0.03 | 8.04 | 0.006*** |
| MD | 0.93±0.05 | 0.90±0.05 | 6.10 | 0.016* |
| RFP |  |  |  |  |
| SLF, FA, L | 0.40±0.02 | 0.42±0.02 | 5.34 | 0.023*** |
| MD, L | 0.75±0.03 | 0.73±0.03 | 6.35 | 0.014*** |
| SLF, FA, R | 0.41±0.02 | 0.42±0.02 | 5.37 | 0.023*** |
| MD, R | 0.76±0.04 | 0.74±0.03 | 6.89 | 0.010*** |

*: *p* < 0.05; ***: *p* < 0.05 after Bonferroni correction. MD：10^-3^mm^2^/s.
